# Preoperative urodynamics and the risk of overdiagnosis and overtreatment of occult urinary incontinence in continent women with advanced pelvic organ prolapse

**DOI:** 10.1007/s00345-026-06404-2

**Published:** 2026-05-08

**Authors:** Susane Mei Hwang, Luis Gustavo Morato de Toledo, Silvia da Silva Carramão, Carolina Remesso Maximo de Jesus, Sarah Zanotto de Carvalho, Antonio Pedro Flores Auge

**Affiliations:** 1 Santa Casa de Sao Paulo School of Medical Sciences, Rua Dr. Cesário Motta Jr., 61, Santa Cecília, São Paulo, SP Brazil; 2 Vila Nova Cachoeirinha Maternity Hospital, São Paulo, SP Brazil

**Keywords:** Suburethral sling, Pelvic organ prolapse, Urodynamics, Urinary incontinence, Stress

## Abstract

**Purpose:**

This study aimed to investigate the impact of preoperative urodynamics (UDS) on the surgical treatment of pelvic organ prolapse (POP). It assessed whether UDS provided additional diagnoses, modified surgical planning, and influenced postoperative outcomes in continent women with advanced POP.

**Methods:**

This retrospective observational study included women with POP stages III–IV, without symptoms of stress urinary incontinence (SUI), who underwent surgical intervention between May 2015 and January 2020.

**Results:**

A total of 226 patients met the inclusion criteria, of whom 102 (45.1%) underwent UDS. Additional diagnoses were identified in 64/102 cases (62.6%), including 21 cases (20.6%) of occult SUI. Surgical planning was modified in 20/102 patients (19.6%), with 18 recommendations for sling placement and two contraindications. The overall rate of occult SUI was 28.8% (65/226). Among 182 patients continent on physical examination (PE), 68 (37.4%) underwent UDS. Postoperatively, the UDS group maintained more pronounced urge incontinence (*p* = 0.006) and storage symptoms (*p* < 0.001) compared to the PE group. No significant difference was observed in the incidence of *de novo* SUI between the UDS group 6/68 (8.8%) and the PE group 11/114 (9.6%) (*p* = 0.853), nor in the need of postoperative sling 4/68 (5.9%) and 5/114 (4.4%), respectively.

**Conclusions:**

Preoperative UDS in women with POP stages III – IV increases diagnostic yield, influences surgical planning, and raises the number of concomitant sling procedures. However, it does not alter postoperative outcomes in patients deemed continent on PE.

## Introduction

One quarter of women without urinary incontinence may develop *de novo* stress urinary incontinence (SUI) after pelvic organ prolapse (POP) surgery [1–3]. This may result from correction of urethral kinking or obstruction following surgical repair of advanced POP [4].

Women with occult SUI are more likely to develop *de novo* SUI after POP surgery [2]. Occult SUI, defined as the stress incontinence observed upon prolapse reduction, can be demonstrated by physical examination (PE) or urodynamics (UDS) [5]. Adding a concomitant midurethral sling in women with occult SUI reduces by 62% the risk of developing *de novo* SUI [6].

However, the investigation of occult SUI lacks standardization regarding reduction techniques and the role of UDS. Some studies use stress test under prolapse reduction at PE [2], whereas others rely on UDS [7]. Previous study showed that PE and UDS are equivalent and yield concordant findings when demonstrating occult SUI using a strictly controlled protocol scenario [8]. Although this study showed equivalence between both methods under controlled conditions, the practical diagnostic value of UDS in advanced POP remains uncertain. Thus, the study aimed to investigate whether preoperative UDS provides additional diagnoses, modifies surgical planning, or affects postoperative outcomes in women with advanced POP and no SUI symptoms.

## Materials and methods

This single-center retrospective observational study included consecutive women with POP stages III–IV [5] undergoing surgery for POP between May 2015 and January 2020. Patients with SUI symptoms or leakage without prolapse reduction were excluded. Preoperative evaluation included clinical history, PE, and stress testing with and without prolapse reduction. Multi-channel UDS was performed at the surgeon´s discretion with no predefined institutional criteria. Urinary symptoms were quantified using the validated Brazilian short form of the International Consultation on Incontinence Questionnaire (ICIQ-SF) [9]. Stress testing during PE was performed with a comfortably full bladder at office evaluation and prolapse reduction with gauze and forceps.

To evaluate the impact of UDS on diagnosis and surgical management, medical records of patients undergoing UDS were reviewed, and results regarding diagnosis and surgical planning were compared before and after the exam. For outcome analysis of UDS impact, only patients without a clear indication for preoperative UDS were included. Therefore, patients with occult SUI on PE were excluded, as they were considered to have complicated SUI (POP + SUI), for which UDS would be already indicated prior to concomitant SUI and POP treatment. The impact of UDS on outcomes was assessed by the incidence of *de novo* SUI and need for a secondary surgical approach for sling or urethrolysis in continent women on preoperative PE under prolapse reduction. *De novo* SUI was diagnosed when patient reported bothersome SUI and had a positive stress test on postoperative follow-up visit at least three months after surgery. POP recurrence was defined when the most distal point exceeded − 1 at any vaginal compartment. Bladder outlet obstruction (BOO) was calculated using the Solomon-Greenwell nomogram [10], with BOO being considered when PdetQmax – 2.2Qmax > 18, and bladder contractility index (BCI) using BCI = PdetQmax + 5Qmax [11].

The study was previously approved by the local ethics committee (registration number: 2.654.203). Urodynamic techniques and terminology followed International Continence Society (ICS) and International Urogynecological Association (IUGA) recommendations [5, 12]. Statistical analysis used IBM SPSS version 25.0. For normally distributed variables, group differences were assessed with Student’s t-test. Non‑normal distributions were analyzed using the Mann–Whitney test. Differences in proportions were evaluated with Pearson’s chi‑square or Fisher’s exact test, adopting a significance level of *p* < 0.05.

## Results

During the study period, 539 women with stage III–IV POP underwent surgery, and 226 met the inclusion criteria. The remaining 233 were excluded for reporting bothersome SUI or leaking without prolapse reduction; 79 discontinued follow-up before three months; and one had cognitive impairment.

**Fig. 1:**
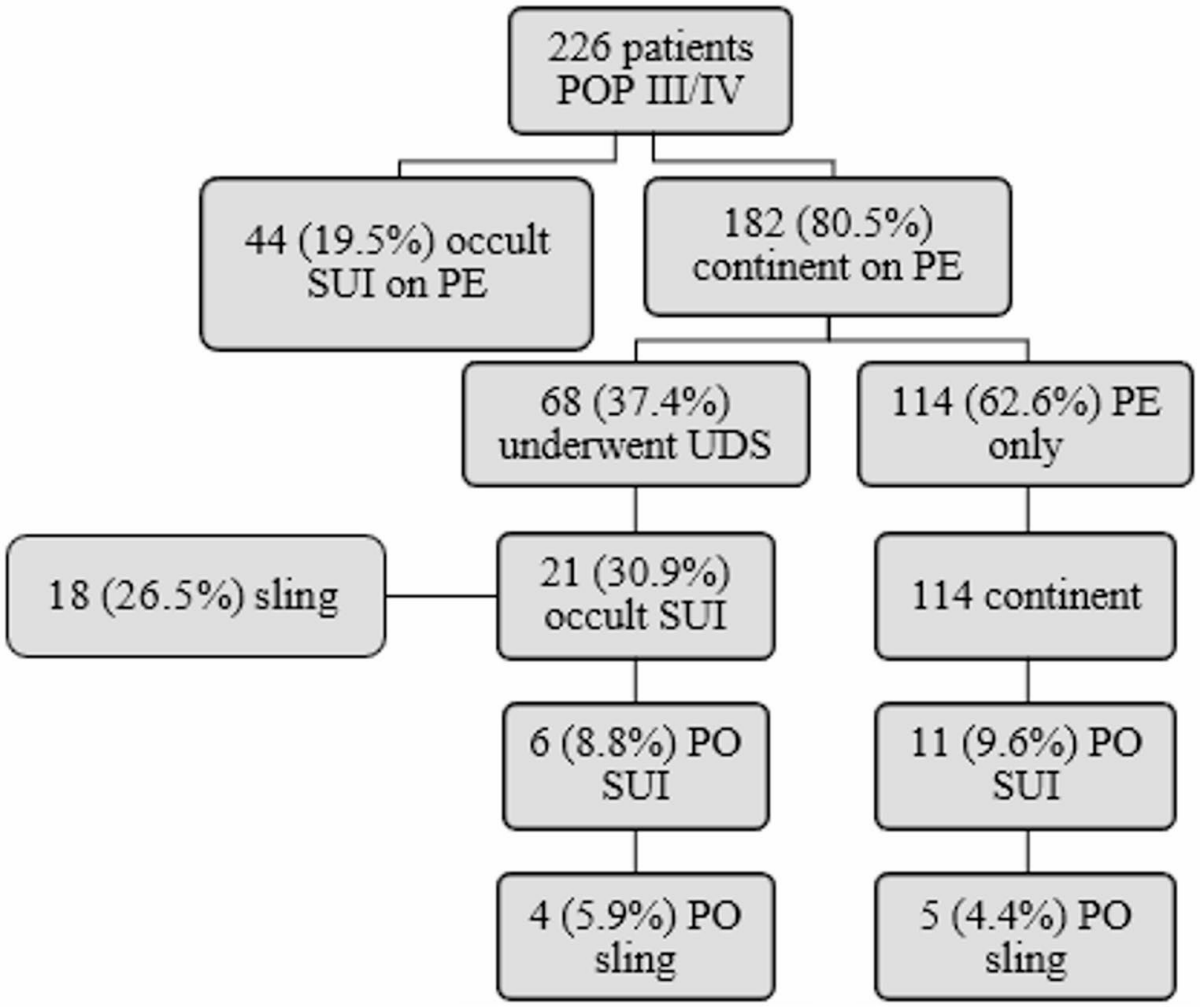
Flowchart of the 226 asymptomatic SUI patients with POP stages III–IV assessed by physical examination and urodynamics. SUI: stress urinary incontinence; PE: physical examination; UDS: urodynamics; PO: postoperative

Baseline demographic and clinical characteristics of the 226 patients, as well as the surgical procedures performed, are presented in Table 1. The overall prevalence of occult SUI identified by PE and/or UDS was 28.8% (65/226). Using PE alone, prevalence was 19.5% (44/226), leaving 182 women classified as continent. Among these, 68 (37.4%) subsequently underwent UDS, as shown in Fig. 1.

**Table 1:** Baseline demographic and clinical characteristics and surgical procedures

	*n* (%)	Mean±(sd)
Age (years)		66.9 (9.2)
BMI (kg/m^2^)		26.7 (4.4)
Parity		3.8 (2.7)
Urinary Symptoms:		
Storage	125 (55.4)	
Voiding	102 (45.1)	
UUI	62 (27.4)	
POP Stage:		
Stage III	143 (63.3)	
Stage IV	83 (36.7)	
Surgical procedures:		
Anterior/apical transvaginal mesh	112 (49.5)	
Colpocleisis	40 (17.7)	
Vaginal hysterectomy	32 (14.2)	
Anterior/ posterior colporrhaphy	25 (11.1)	
Laparoscopic sacrocolpopexy	9 (4.0)	
Other	8 (3.5)	

*n* number; sd: standard deviation; BMI: body mass index; UUI: urgency urinary incontinence; POP: pelvic organ prolapse

UDS was performed in 102 women (45.1%), and occult SUI identified in 47.1% (48/102). UDS provided at least one additional diagnosis in 64 cases (62.7%), including 21 cases (20.6%) of occult SUI. The main UDS findings are summarized in Table 2. Eight patients (7.8%) were unable to void during the pressure–flow study. Based on UDS results, the planned surgical procedure was modified in 20 of 102 cases (19.6%), including 18 recommendations for sling placement and two contraindications.

**Table 2:** Urodynamic findings

	*n*	%
Post void residual volume ≥ 100 ml	21/102	20.5
Detrusor overactivity	10/102	9.8
Mean VLPP (cmH_2_O)	67.7	-
Weak BCI (≤ 100)	46/102	45.1
Normal BCI (> 100 and < 150)	34/102	33.3
Strong BCI (≥ 150)	14/102	13.7
UDS Occult SUI	48/102	47.1
Bladder outlet obstruction	10/102	9.8
At least one urodynamic diagnosis added	64/102	62.7

n: number; ml: milliliter; VLPP: Valsalva leak point pressure; BCI: bladder contractility index; UDS: urodynamics; SUI: stress urinary incontinence

The 182 continent women assessed by PE were analyzed separately and divided into two groups: the PE group (occult SUI evaluated only by PE) and the UDS group (evaluated by both PE and UDS). Table 3 shows their preoperative characteristics. Women in the UDS group reported more storage symptoms, UUI, and voiding symptoms than those in the PE group. UDS additionally identified occult SUI in 30.9% (21/68).

**Table 3:** Comparison of preoperative baseline characteristics of 182 continent patients on physical examination

	UDS (*n* = 68) *n* (%)		PE (*n* = 114) *n* (%)	*P* value
Mean age (years)	64.0		66.3	0.104^a^
Mean BMI	26.4		26.8	0.905^b^
Mean parity	3.9		3.6	0.651^b^
Previous POP surgery	9 (13.2)		19 (16.7)	0.535^c^
Previous histerectomy	5 (7.3)		13 (11.4)	0.376^c^
Anticholinergic	1 (1.5)		2 (1.8)	1.000^d^
Preoperative Symptoms:				
UUI	26 (38.2)		20 (17.5)	**0.002** ^c^
Storage symptoms	48 (70.6)		53 (46.5)	**0.002** ^c^
Voiding symptoms	37 (54.4)		42 (36.8)	**0.021** ^c^
Dyspareunia	5 (7.3)		2 (1.7)	0.157^c^
POP-Q:				
Ba (mean)	+ 4.0		+ 3.7	0.852^b^
Bp (mean)	+ 0.7		+ 1.1	0.236^b^
C (mean)	+ 2.6		+ 2.7	0.599^b^
TVL (mean)	8.3		7.9	0.064^b^
POP-Q stage:				
Stage III	47 (69.1)		77 (67.5)	0.826^c^
Stage IV	21 (30.9)		37 (32.5)	
UDS Occult SUI	21 (30.9)		-	**< 0.001** ^c^

UDS: urodynamics; PE: physical examination; n: number; BMI: body-mass index (the body-mass index is the weight in kilograms divided by the square of the height in meters); POP: pelvic organ prolapse; UUI: urgency urinary incontinence; POP-Q: pelvic organ prolapse quantification system; Point Ba: the most distal position of the anterior vaginal wall; Point Bp: the most distal position of the posterior vaginal wall; Point C: the most distal position of the cervix or the vaginal cuff; TVL: total vaginal length; SUI: stress urinary incontinence; a: Student’s t-test; b: Mann-Whitney test; c: Chi-square test; d: Fisher’s Exact test

Postoperatively, the UDS group continued to show higher rates of UUI and storage symptoms than the PE group, although the incidence of *de novo* SUI was similar between groups (Table 4). A greater proportion of women in the UDS group received a concomitant midurethral sling (26.5% vs. 0%, *p* < 0.001). Nevertheless, rates of *de novo* SUI (8.8% vs. 9.6%) and the need for postoperative sling placement (5.8% vs. 4.4%) were comparable. Among the four cases of postoperative sling in the UDS group, three were continent upon UDS and one occult SUI did not undergo concomitant sling along with the first prolapse surgery and had an early prolapse recurrence, requiring a second surgery for POP and sling. Cure rates in the sling group were 73.1% (19/26) for UUI, 64.6% (31/48) for storage symptoms, and 91.9% (34/37) for voiding symptoms, compared with 100% (20/20), 90.6% (48/53), and 100% (42/42), respectively, in the PE group.

**Table 4:** Comparison of intraoperative and postoperative outcomes between UDS and PE group

	UDS (*n* = 68) n (%)	PE (*n* = 114) n (%)	*P* value
Midurethral sling	18 (26.5)	-	< 0.001^a^
Intraoperative adverse events	1 (1.5)	2 (1.8)	1.000^b^
Bladder catheterization > 48 h	2 (3.0)	2 (1.8)	0.856^a^
Mean follow-up (months)	15.6	13.6	0.522^c^
SUI symptom	**8 (11.8)**	**15 (13.2)**	**0.784** ^**a**^
UUI symptom	10 (14.7)	4 (3.5)	**0.006** ^**a**^
Storage symptom	20 (29.4)	9 (7.9)	**< 0.001** ^**a**^
Voiding symptom	5 (7.4)	2 (1.8)	0.104^b^
*De novo* UUI	3/42 (7.1)	4/94 (4.2)	0.481^a^
*De novo* storage symptom	3/20 (15.0)	4/61 (6.5)	0.244^a^
*De novo* voiding symptom	2/31 (6.5)	2/72 (2.8)	0.582^b^
Positive stress test	**6 (8.8)**	**11 (9.6)**	**0.853** ^**a**^
Vaginal mesh exposure	3 (4.4)	4 (3.5)	1.000^b^
Physical therapy	8 (11.8)	12 (10.4)	0.796^a^
Anticholinergic	5 (7.4)	4 (3.5)	0.297^b^
POP recurrence	17 (25.0)	33 (28.9)	0.564^a^
Reoperation:	6 (8.8)	10 (8.8)	
Sling	**3 (4.4)**	**5 (4.4)**	
POP	-	4 (3.5)	
POP + sling	**1 (1.4)**	**-**	
Urethrolysis	1 (1.4)	0	
Resection of mesh extrusion	1 (1.4)	1 (0.9)	0.991^a^

UDS: urodynamics; PE: physical examination; SUI: stress urinary incontinence; UUI: urgency urinary incontinence; POP: pelvic organ prolapse; n: number. a: Chi-square test; b: Fisher’s Exact test; c: Mann-Whitney test

Two intraoperative adverse events occurred in the PE group: one major bleeding during sacrospinous ligament dissection, managed with a vaginal pack, and one bladder perforation during laparoscopic sacrocolpopexy. All mesh exposures were related to POP surgery. In the UDS group, two women required prolonged catheterization.

## Discussion

Although preoperative UDS offers additional information on bladder and urethral function, most of lower urinary tract symptoms (LUTS) resolve after POP repair [1, 13]. In this study, UDS contributed at least one additional diagnosis in 64/102 patients (62.7%), including occult SUI in 21/102 (20.6%). As a result, the surgical plan was modified in 19.6%, all involving continence procedures (90% sling indications and 10% contraindications).

Glass et al. [14] analyzed 316 stress-continent women who underwent UDS before POP surgery and found that only 3.5% required changes in management based on UDS. Among those evaluated specifically for occult SUI, however, the condition was detected in 29.4%, leading to sling placement in 82% of this subgroup. Another study of 118 women with stage III–IV POP reported that preoperative UDS altered medical or surgical management in 19.5% of cases and informed preoperative counseling in 56% [15].

When comparing surgical outcomes of PE continent patients with and without UDS, no differences were observed in the incidence of *de novo* SUI (8.8% in the UDS group vs. 9.6% in the PE group, *p* = 0.853) or in the need for additional surgery for SUI (5.8% vs. 4.4%, *p* = 0.991). Although women who did not undergo UDS had fewer diagnoses of occult SUI and received fewer concomitant slings, they did not experience higher rates of postoperative *de novo* SUI, contradicting the expectation that fewer slings would translate into more postoperative leakage. These findings suggest that performing UDS in PE continent patients without a clear indication may lead to overdiagnosis of occult SUI and unnecessary sling placement.

Schierlitz et al. [7] randomized 80 patients with occult SUI to concomitant midurethral sling or no sling and performed UDS six months after prolapse surgery. Postoperative UDS identified *de novo* SUI in 15% of the sling group and 66% of the no‑sling group. Despite these findings, all women in the sling group and 81% in the no‑sling group reported no incontinence symptoms. These results suggest that UDS may overdiagnoses SUI, as 80% of patients were asymptomatic despite positive findings. Other authors have proposed that abdominal pressures generated during UDS may exceed those typically experienced by older women in daily life, potentially limiting the test’s real‑world applicability [16].

Espuña-Pons et al. [17] showed that bladder volume during stress testing influences the detection of occult SUI, with higher positivity rates at intravesical volumes above 200 ml. In our study, PE stress testing was performed with a naturally full bladder, without measuring volume. Had we retrogradely filled the bladder to 300 ml, more cases of occult SUI might have been detected, potentially increasing the number of concomitant slings.

Women with uncomplicated SUI do not require preoperative UDS before surgery [18, 19]. Occult SUI, however, cannot be considered uncomplicated because it occurs in the context of POP and is frequently accompanied by voiding symptoms. Accordingly, women with occult SUI on PE should be classified as having complicated SUI, supporting the indication for preoperative UDS. For this reason, patients with occult SUI on PE were excluded from the surgical outcomes analysis.

The role played by UDS in stress continent women with POP it´s still very debatable. The American Urological Association (AUA) suggests that UDS can be indicated before treating women with high-grade POP if SUI has not been demonstrated by POP reduction upon PE and women with significant voiding disfunction or elevated post-void residual [20]. The European Association of Urology (EAU) recommends performing POP reduction test (either upon PE or UDS) in continent women to identify occult SUI for preoperative counseling [21]. Risks and benefits must be balanced while considering not only that UDS is an invasive test and there exists morbidity associated therewith, but also that it can possibly result in unnecessary slings being performed. On the other hand, UDS may have an important role in counselling the patient regarding outcomes and possible complications, also from a medico-legal viewpoint.

It is important to note that although patients who underwent UDS reported a higher burden of urinary symptoms, none of them presented with SUI on PE during routine office evaluation prior to UDS. Thus, while symptom severity may have influenced the physician’s decision to request UDS, these patients shared the common characteristic of absence of clinically demonstrable SUI. Due to the retrospective nature of the study, predefined criteria for UDS referral could not be applied, and this potential selection bias could not be fully controlled; therefore, the results should be interpreted in this context. Well-designed prospective studies are needed to further clarify the role of UDS in continent women undergoing POP surgery.

## Conclusions

Preoperative UDS in women with POP stages III–IV increases diagnostic yield, influences surgical planning, and raises the number of concomitant sling procedures. However, it does not alter postoperative outcomes in patients deemed continent on PE.

## Data Availability

The data that support the findings of this study are available from the corresponding author upon reasonable request.

## Abbreviations

POP: Pelvic organ prolapse
SUI: Stress urinary incontinence
UDS: Urodynamics
PE: Physical examination
ICIQ-SF: International Consultation on Incontinence Questionnaire
BOO: Bladder outlet obstruction
BCI: Bladder contractility index
BMI: Body mass index
UUI: Urgency urinary incontinence
LUTS: Lower urinary tract symptoms
